# 2-Hydroxy-4-methoxybenzaldehyde (HMB) disrupts ergosterol biosynthesis, redox metabolism, and DON biosynthesis of *Fusarium graminearum* revealed by transcriptome analysis

**DOI:** 10.3389/fmicb.2025.1514170

**Published:** 2025-06-02

**Authors:** Hongying Xiao, Yiming Zhang, Qian Li

**Affiliations:** Henan Key Laboratory of Cereal and Oil Food Safety and Nutrition, College of Food Science and Engineering, Henan University of Technology, Zhengzhou, China

**Keywords:** plant-derived natural compound, HMB, ergosterol biosynthesis, oxidative stress response, mycotoxin regulation, transcriptome analysis

## Abstract

The phytopathogenic fungus *Fusarium graminearum* causes Fusarium head blight, which threatens agricultural yield and human health. We previously demonstrated that a plant-derived natural compound, 2-hydroxy-4-methoxybenzaldehyde (HMB), can inhibit *F. graminearum*. This study continued to investigate its antifungal mechanism. Compared to the control, HMB treatment at the minimum inhibitory concentration (MIC) significantly reduced ergosterol levels by 61.78%, indicating compromised fungal membrane integrity. Concurrently, intracellular reactive oxygen species (ROS) levels increased 22-fold, accompanied by a 146.03% increase in hydrogen peroxide (H₂O₂) content. Meanwhile, superoxide dismutase (SOD) activity increased, while catalase (CAT) activity declined, suggesting a marked change of redox metabolism upon HMB exposure. In addition, Quantitative Real-time PCR (qRT-PCR) analysis revealed that HMB treatment significantly regulated the expression of genes participating in ergosterol biosynthesis (*Erg2*, *Erg5*, *Erg6*, etc.), DON biosynthesis (up to 16 genes), the redox system (*MnSOD*, *Cu/ZnSOD*, *GSS*, and *CAT*), global regulators (*LaeA*, *VeA*, and *VelB*), and stress signaling pathways (*Hog1*, *Ssk1*, *Ssk2*, and *Pbs2*). These findings revealed the in-depth antifungal mechanism of HMB and proposed that HMB holds potential as an antifungal agent.

## 1 Introduction

*Fusarium graminearum* is a member of the phylum Ascomycota and is an important phytopathogenic fungus (Lee et al., 2009). It can infect various crops such as wheat, corn, barley, and rice, causing a spectrum of destructive diseases such as ear rot disease, gibberella stalk rot, and Fusarium head blight (FHB) (Ferrigo et al., 2016). Among the diseases, FHB poses significant threats to global food security (Ji et al., 2019). Severe FHB epidemics occur at least once every 4 or 5 years in countries such as China, the United States, and the European Union (Figueroa et al., 2017). From 2000 to 2018 in China, FHB affected over 4.5 million hectares of wheat fields each year, covering about 20% of the total planted area and causing annual yield losses exceeding 3.41 million metric tons (Chen et al., 2019). FHB outbreaks in 2015–2016 caused losses exceeding 1.4 billion dollars in the US (Wilson et al., 2018).

*F. graminearum* can also produce toxic secondary metabolites, including deoxynivalenol (DON), zearalenone (ZEN), and nivalenol (NIV) (Feizollahi and Roopesh, 2021; Häggblom and Nordkvist, 2015; Podgórska-Kryszczuk et al., 2022). Among these metabolites, DON (also known as vomitoxin) is the most prevalent toxin. When DON accumulates in contaminated crops, it can enter the food supply through unprocessed agricultural products during transportation and storage. Alarmingly, DON persists even after processing due to its heat-resistant nature, making it difficult to remove. As DON primarily affects the gut, immune system, and brain function, exposure to it could lead to intestinal irritation, vomiting, anorexia, weight loss, and immune suppression, posing significant risks to both human and animal health (Payros et al., 2016; Pestka, 2010). Therefore, controlling the growth of *F. graminearum* and the biosynthesis of DON is crucial for both crop production and food safety.

Currently, chemical fungicides (e.g., carbendazim, tebuconazole, and metconazole) are ubiquitously applied (Diao et al., 2018; Mendes et al., 2018). However, the emergence of resistant strains and the negative impacts on the environment and human health are serious problems thereof (Sun et al., 2018). Subsequently, recent studies have switched from chemical fungicides to plant-derived natural compounds, as they are rich in resources, safe for human and animals, environmentally friendly, and cost-effective, etc. Some natural compounds (e.g., glabridin, ferulic acid, myrcene, perillaldehyde, and myriocin) exhibit remarkable inhibitory effects against *F. graminearum* in recent published papers (Albayrak et al., 2023; Meng et al., 2024; Shao et al., 2021; Yan et al., 2023; Yang et al., 2021). Notably, cell membranes are common antifungal targets for the above-mentioned compounds.

2-Hydroxy-4-methoxybenzaldehyde (HMB) is an active compound in many herbals, such as *Decalepis hamiltonii*, *Hemidesmus indicus*, *Mondia whitei*, *Periploca sepium*, and *Sclerocarya caffra* (Subban and Mohan, 2003). It exhibits excellent antimicrobial, anti-inflammatory, hepatoprotective, and neuroprotective activities (Rathi et al., 2017). In particular, the antimicrobial spectrum of HMB is broad, including *Escherichia coli*, *Staphylococcus epidermidis*, *Candida albicans*, and *Helicobacter pylori* (Srikanta et al., 2011; Wang et al., 2010). Our research indicates that HMB is a more effective antifungal compound in comparison with its derivatives vanillin and *o*-vanillin against both *Aspergillus flavus* and *F. graminearum* (Li et al., 2024). Besides, compared to other compounds, the antifungal effect of HMB against *F. graminearum* is stronger than myrcene (MIC: 25 μg/μL) (Albayrak et al., 2023), perillaldehyde (MIC: 240 μg/L) (Meng et al., 2024), and similar to myriocin (MIC: 64 μg/mL) (Shao et al., 2021). Nevertheless, the mechanism of HMB against *F. graminearum* has not been thoroughly studied yet. In the present work, the ergosterol content was first determined. Then, we quantified the reactive oxygen species (ROS) production and the activities of related enzymes, as well as intermediate products within redox system. Moreover, in combination with transcriptome analysis and Quantitative Real-time PCR (qRT-PCR), relative expressions of important genes were quantified. This study provides in-depth insights in the antifungal and anti-mycotoxigenic mechanism of HMB, especially the relationship between redox system and DON biosynthesis. Meanwhile, the potential antifungal target(s) can also be identified and screened for effective controlling of *F. graminearum* in agricultural production and food preservation.

## 2 Materials and methods

### 2.1 Media and strains

*Fusarium graminearum* PH-1 was cultured on Potato Dextrose Agar (PDA), which was formulated with 20% (w/v) potato, 0.2% (w/v) dextrose, and 2% (w/v) agar at 28 ± 2°C. For the present study, mycelia were collected from Potato Dextrose Broth (PDB), a medium prepared with 2% (w/v) potato and 0.2% (w/v) dextrose. Five plugs of *F. graminearum* were cultured in 100 mL of carboxymethylcellulose sodium medium (CMC, 1.5% (w/v) carboxymethylcellulose sodium, 0.1% (w/v) yeast extract, 0.1% (w/v) NH₄NO₃, 0.1% (w/v) KH₂PO₄, and 0.05% (w/v) MgSO₄·7H₂O) for spore collection.

### 2.2 Ergosterol content determination

A suspension of 10^4^ spores was added into 50 mL of PDB and incubated under shaking conditions (150 rpm) for 36 h. Subsequently, HMB (98%, CAS: 673–22-3) was supplemented into the cultures to achieve final concentrations of 0 (control), 1/4 MIC (50 μg/mL), 1/2 MIC (100 μg/mL), and MIC (200 μg/mL), as determined previously (Li et al., 2024). The treated cultures were further incubated for 36 h. Ergosterol extraction and quantification were performed according to the method described by Khan et al. (2010). Briefly, the harvested mycelia were ground by liquid nitrogen, 20 mg of mycelia were mixed with 5 mL of 25% (w/v) potassium hydroxide alcohol. The mixture was vortexed for 10 min and then heated at 85°C for 2 h. After cooling, N-heptane (3 mL) and sterile water (1 mL) were included. The biphasic system was vortexed for an additional 10 min and subsequently stored at −20°C overnight to facilitate phase separation. Absorbance of the upper phase were assayed at wavelengths of 230 nm and 282 nm with a UV-6100S double beam spectrophotometer (Shanghai Mapada Instruments Co., Ltd., China), and the ergosterol levels were then determined using the formula below:


$$\text{Ergosterol}\;(\%)=\frac{\left(A_{282}/290-A_{230}/518\right)}{\text{lyophilized mycelia weight}}$$

where the E (%/cm) values of crystalline ergosterol and dehydroergosterol were 290 and 518, respectively.

### 2.3 ROS content determination

ROS content was evaluated using an assay kit (S0033S) from Beyotime Biotechnology (Shanghai, China). In short, the mycelia were treated with the reactive oxygen fluorescent probe (DCFH-DA) for 30 min, followed by observation with a fluorescence microscope (Leica Microsystems GmbH, Germany). Fluorescence intensity was subsequently assayed using ImageJ (v1.54, National Institutes of Health (NIH), Bethesda, MD, United States).

### 2.4 Determination of superoxide dismutase (SOD), catalase (CAT) and glutathione peroxidase (GPx) activity

After grinding the mycelia in liquid nitrogen, 500 mg of the sample was suspended in phosphate buffered saline (PBS) (5 mL). The suspension was centrifuged at 12,000 rpm for 10 min at 4°C, and the supernatant was used as the crude enzyme extract for testing. SOD activity was assessed using a previously established method (Sahin et al., 2018) with minor modifications. The supernatant (50 μL) was thoroughly blended with PBS (4.5 mL), nitrotetrazolium blue chloride (0.3 mL), 220 mM methionine (0.3 mL), and 33 μM riboflavin (0.3 mL). After illumination at 4000 lux for 20 min at 25°C, the fluorescence intensity of the mixture was quantified at a wavelength of 560 nm. The formula below was utilized to determine SOD activity:


$$\text{SOD}\;\text{activity}\;(U/g\;\text{FW})=\frac{(A_{1}-A_{2})\times V_{t}}{A_{1}\times W\times V_{s}\times 0.5}$$

where A_1_ and A_2_ are the absorbance values at 560 nm for the control and sample groups, respectively. W (g) is the fresh weight of the mycelia, V_t_ (mL) represents the total volume of the enzyme extract, and *V_s_* (mL) corresponds to the volume of enzyme solution used in the assay. The method for measuring CAT activity was as earlier established (Valenzuela- Cota et al., 2019). One hundred microliters of the crude enzyme extract were mixed with 1 mL of 0.3% (w/v) hydrogen peroxide (H₂O₂) and 2 mL of PBS. Subsequently, the absorbance was measured at a wavelength of 240 nm every minute. The method for calculating CAT activity was outlined as follows:


$$\text{CAT}\;\text{activity}\;(U/g\;\text{FW})=\frac{V_{t}\times \Delta A_{240}}{V_{s}\times W\times 0.01\times t}$$

where *V_s_* (mL) and V_t_ (mL) refer to the enzyme solution and crude enzyme extract volumes, respectively. ΔA240 indicates the variation in absorbance at 240 nm per minute, and the mycelial weight and reaction time are represented by W (g) and t (min), respectively. The kit from Nanjing Jiancheng Bioengineering Institute (NJBI) (H545-1-1) was used to assay the GPx activity.

### 2.5 H_2_O_2_ and glutathione (GSH) content determination

The H_2_O_2_ content was assessed by a commercial kit (BC3590) from Beijing Solarbio Science & Technology Co., Ltd. The GSH content was evaluated following a previously established method (Gill et al., 2012). One milligram of mycelia was mixed with 3 mL of 5% metaphosphoric acid, then centrifuged at 12,000 rpm for 10 min at 4°C. Subsequently, 1 mL of the supernatant was transferred to a solution containing 1 mL of PBS and 0.5 mL of 5,5’-Dithiobis-(2-nitrobenzoic acid) (DTNB), followed by incubation for 20 min at room temperature. The absorbance was measured at a wavelength of 412 nm. A standard curve was generated using GSH concentrations ranging from 0 to 50 μg/mL.

### 2.6 Transcriptome analysis

#### 2.6.1 Library construction and sequencing

RNA was extracted from mycelia treated with 0 and 1/2 MIC (100 μg/mL) of HMB for 36 h using an RNA extraction kit (RC411-01, Vazyme Biotech, Inc., China). The RNA samples were then subjected to transcriptome sequencing by the Beijing Genome Institute (BGI). For mRNA enrichment from total RNA, poly-A mRNA was captured using Oligo (dT) magnetic beads. The mRNA was fragmented with a buffer, reverse-transcribed into cDNA using random N6 primers, and extended into double-stranded cDNA. The cDNA was end-repaired, 5′-phosphorylated, and a single ‘A’ overhang was added to the 3′ end. An adapter with a 3′ ‘T’ overhang was ligated. The product was PCR-amplified, denatured to single-stranded DNA, and circularized via a bridge primer, forming a single-stranded DNA library. The library was sequenced on an Illumina NovaSeq 6,000 platform to obtain raw sequencing data.

#### 2.6.2 Bioinformatics analysis

The sequencing data were analyzed and filtered using SOAPnuke software (v1.5.6) (Chen et al., 2018). The cleaned data was conducted by the Dr. Tom multi-omics data mining system.^1^ The genome of *F. graminearum* PH-1 (NCBI accession number: GCF_000240135.3_ASM24013v3) was used as the reference, and comparative analysis was conducted with HISAT2 (Johns Hopkins University, USA) (Kim et al., 2015), which employs a hierarchical indexing approach for aligning spliced transcripts. Bowtie2 was adopted to map the clean data to comprehensive gene set, including both non-coding and known and novel coding transcripts (Langmead and Salzberg, 2012). To obtain a more comprehensive understanding of gene functions associated with phenotypic changes, gene expression levels were assessed using RSEM software (v1.3.1) (Li and Dewey, 2011), and hypergeometric tests were employed for GO and KEGG analysis.

### 2.7 qRT-PCR analysis

Reverse transcription and qRT-PCR were performed using the R433 and Q712 kits from Vazyme Biotech Co. Ltd. (Nanjing, China). Primers (sequences are in Supplementary Table S1) used in this experiment were designed and synthesized by Sangon Biotech Co. Ltd. (Shanghai, China). *β*-Tubulin was used as the control gene for data normalization, and the relative gene expression levels were calculated using the 2^-ΔΔCT^ method.

### 2.8 Statistical analysis

The experiments were conducted at least three times in triplicate. The quantified results were presented as the mean ± SD (mean ± standard deviation) and the significance for ergosterol content, ROS fluorescence intensity, enzyme activities, as well as GSH and H₂O₂ levels was calculated by one-way ANOVA (*p* < 0.05) test. Statistical analysis of the qRT-PCR data was conducted using Student’s t-test with a significance threshold set at *p* < 0.05, and all analyses were performed via SPSS 27 (IBM, Armonk, NY, USA).

## 3 Results

### 3.1 HMB decreased ergosterol content

**Figure 1:**
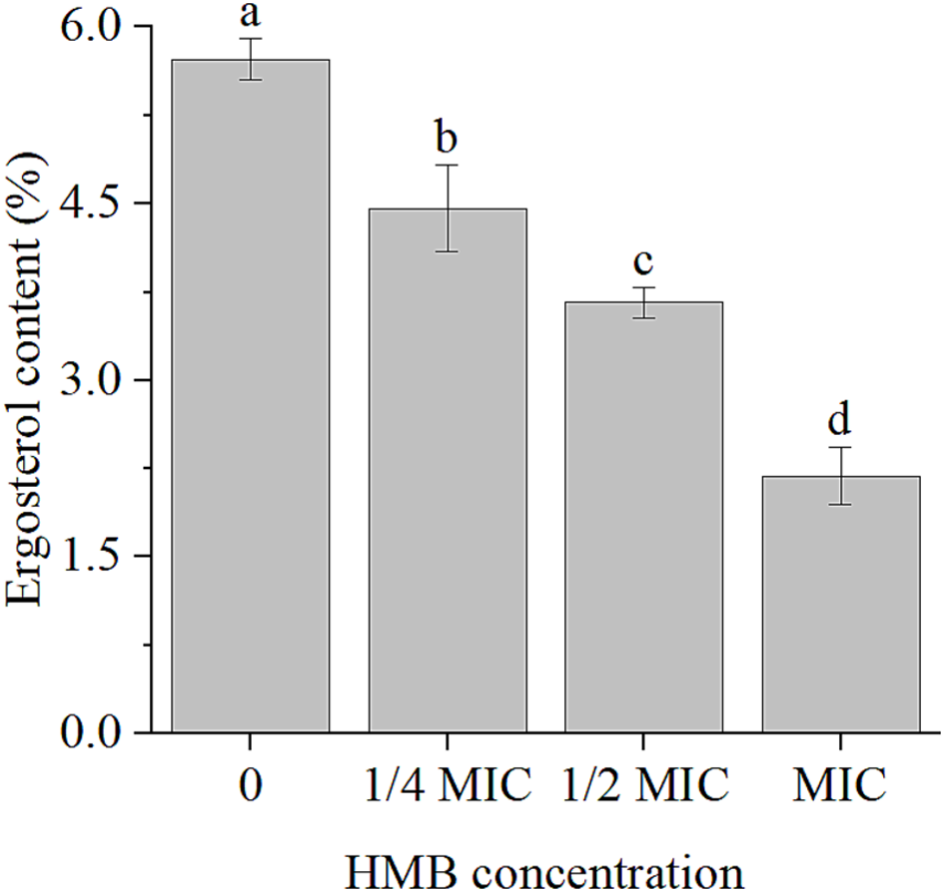
Content of ergosterol in *F. graminearum* treated with HMB at 0, 1/4, 1/2 and MIC. HMB: 2-Hydroxy-4-methoxybenzaldehyde; MIC: minimum inhibitory concentration. a-d significant difference (*p* < 0.05) according to Duncan’s multiple range test.

As the primary sterol molecule in fungal cell membranes, ergosterol not only contributes to the fluidity, permeability, and stability of cell membranes, but also plays important roles in various cellular processes, such as nutrient uptake, signal transduction, and ion transport (Jordá and Puig, 2020). As treatment with HMB disrupted the cell membrane integrity of *F. graminearum* (Li et al., 2024), we quantified ergosterol content. As illustrated in Figure 1, in comparison with the control group, treatment with different concentrations of HMB (1/4MIC, 1/2MIC, and MIC) reduced the content by 22.16, 36.13, and 61.78%, respectively.

### 3.2 HMB disturbed redox metabolism of *F. graminearum*

**Figure 2:**
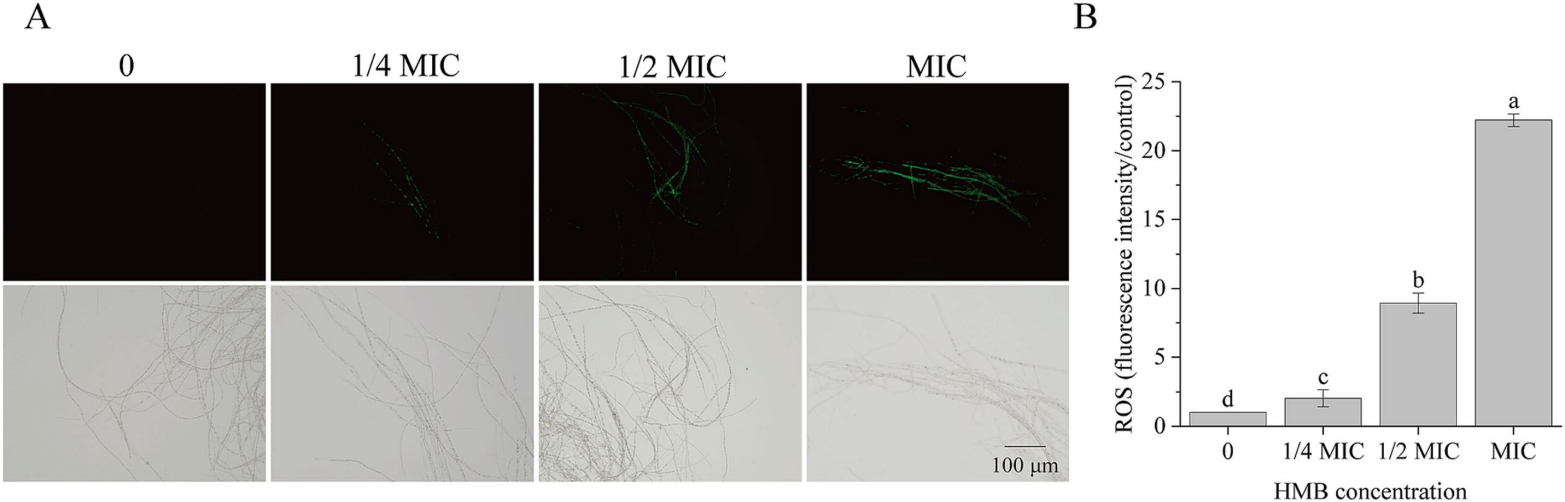
Influence of HMB (0, 1/4 MIC,1/2 MIC, and MIC) on ROS production. **(A)** Mycelia staining with CM-H2DCFDA (upper row: fluorescence images; lower row: bright field images); **(B)** Fluorescence intensity of ROS. a-c significant difference (*p* < 0.05) according to Duncan’s multiple range test.

As a typical product of lipid peroxidation, malondialdehyde (MDA) is commonly used as an indicator of oxidative damage to cell membranes. In our recent work, elevation of MDA content suggested that HMB disrupted lipid peroxidation in cell membranes (Li et al., 2024), leading to the assumption that the redox system was perturbed. To investigate this, we first examined whether HMB treatment leads to intracellular ROS accumulation. As shown in Figure 2, mycelial fluorescence intensity increased significantly with higher HMB concentrations (Figure 2A). Quantitative analysis showed that fluorescence intensities in the 1/2 MIC and MIC groups were approximately 9- and 22-fold higher, respectively, than those in the control (Figure 2B). These results demonstrate that HMB treatment triggers ROS accumulation in *F. graminearum* mycelia.

**Figure 3:**
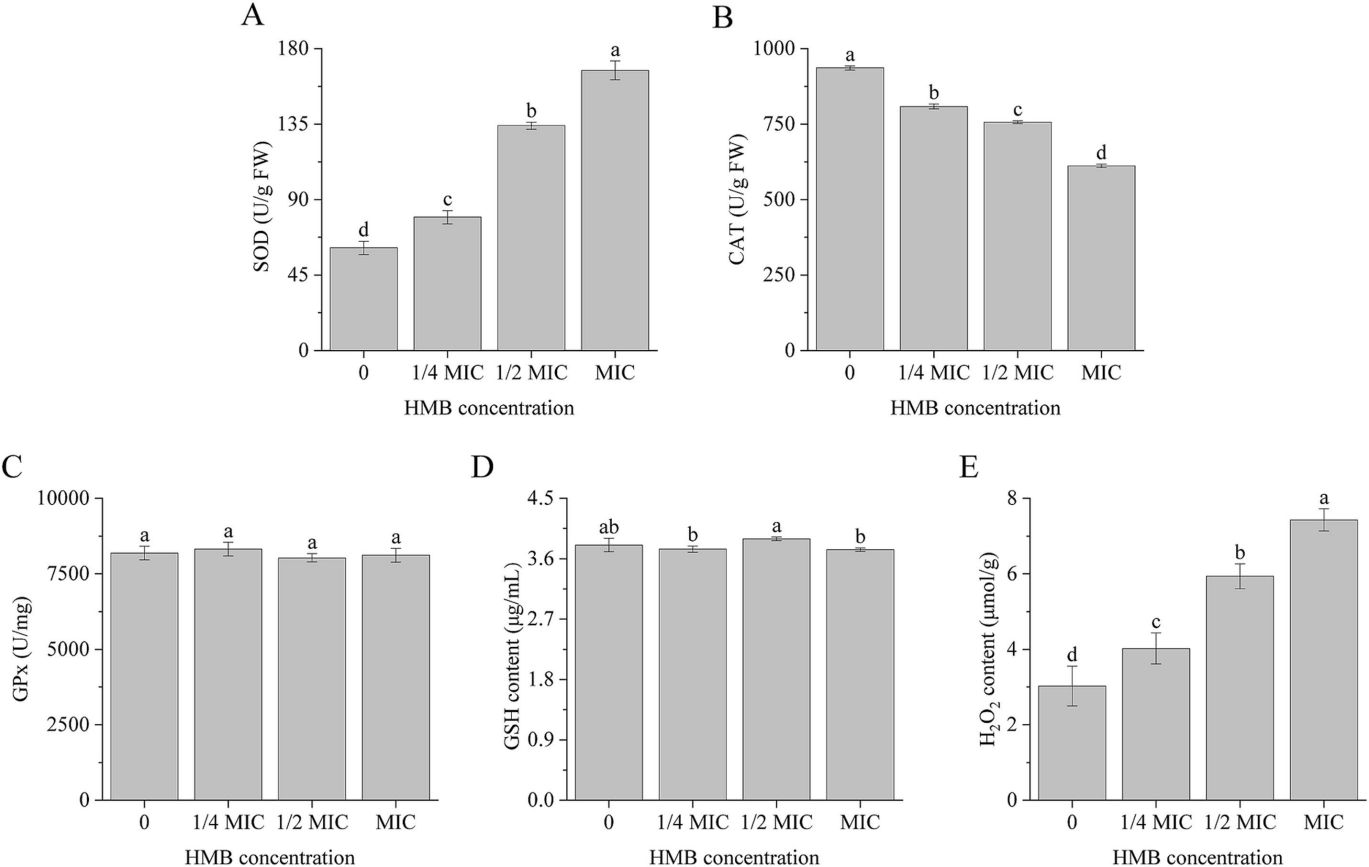
Activities of SOD **(A)**, CAT **(B)**, GPx **(C)**, and content of GSH **(D)** and H_2_O_2_ **(E)**. a-d significant difference (*p* < 0.05) according to Duncan’s multiple range test.

Organisms have developed well-established mechanisms to counteract oxidative stress by eliminating elevated levels of cellular ROS. These mechanisms are generally classified into enzymatic and non-enzymatic systems (Koubaa and Brini, 2020). The enzymatic system contains CAT, SOD, peroxiredoxin, and GPx, and non-enzymatic system contains GSH, ascorbic acid, polyamines, flavonoids, alkaloids, and carotenoids (Heller and Tudzynski, 2011; Jamieson, 1998). As the first line of defense, SODs convert superoxide radicals (O_2_^−^) to H_2_O_2_, which is subsequently detoxified into water (H_2_O) by GPx and CAT (Fridovich, 1995; Heller and Tudzynski, 2011). To investigate the perturbation of the redox system in response to ROS accumulation, we measured the activities of SOD, CAT, and GPx, as well as the levels of GSH and H_2_O_2_. As illustrated in Figure 3A, SOD activity was elevated by 29.82, 118.82, and 172.52% in the 1/4 MIC, 1/2 MIC, and MIC groups, respectively. Meanwhile, the CAT activities were reduced by 13.64, 19.18, and 34.65%, respectively (Figure 3B). However, the GPx activity remained unchanged, even at the MIC group (Figure 3C). In combination with the catalyzing of GPx, GSH can be oxidized to oxidative glutathione (GSSG) during oxidative stress defense (Traynor et al., 2019). In this experiment, the GSH content did not show any significant variations (Figure 3D). However, in comparison with the control group, H₂O₂ content exhibited marked increases of 33.11, 96.36, and 146.03% for the 1/4 MIC, 1/2 MIC, and MIC groups, respectively (Figure 3E).

### 3.3 RNA-seq analysis

**Figure 4:**
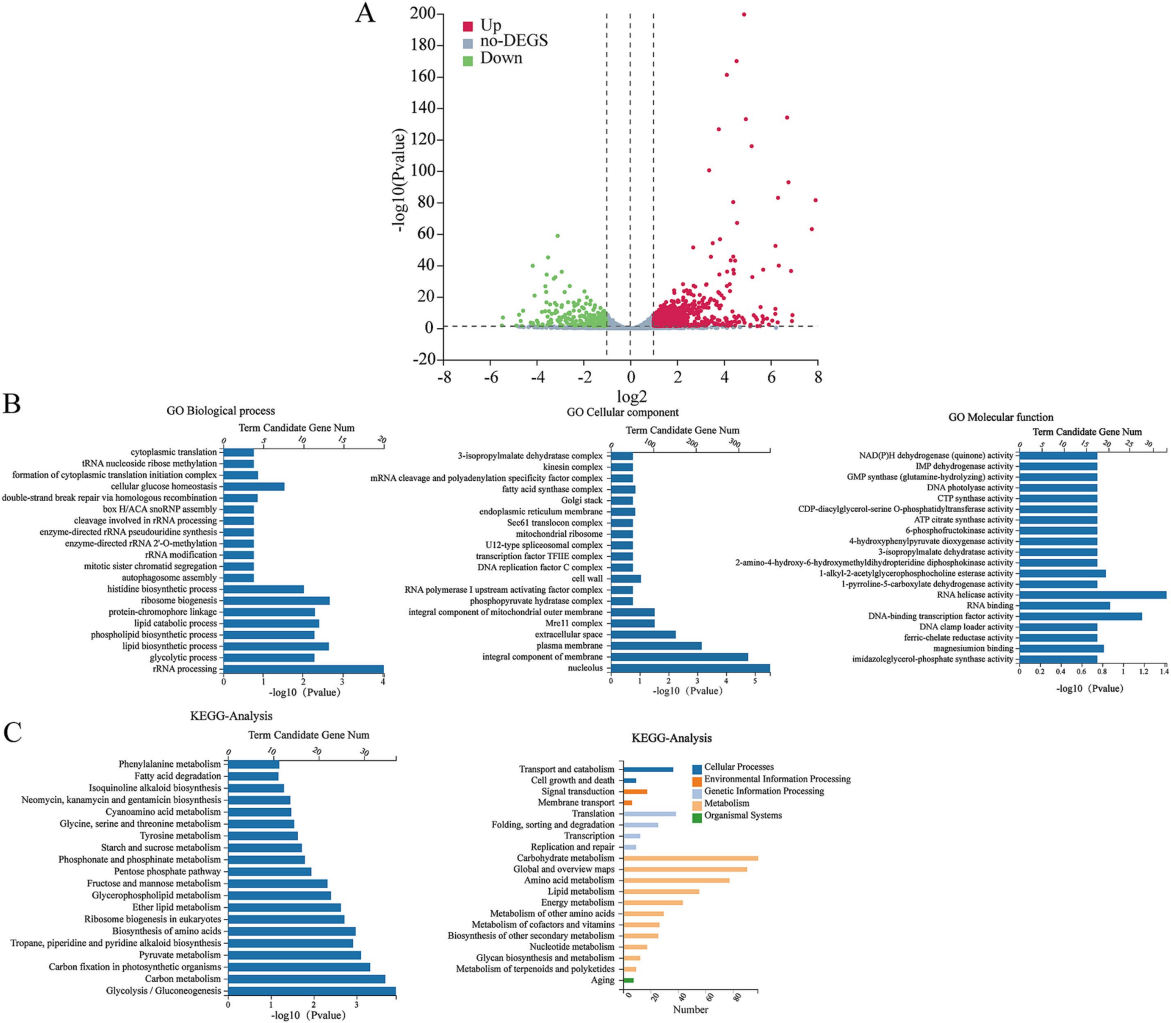
Volcano plots of DEGs after treatment of HMB at 1/2 MIC **(A)**: The red dots and green dots represent up-regulated and down-regulated DEGs, respectively, and the grey dots represent genes with no significant changes in expression. Functional categorization of Biological Process (BP), Cell Components (CC), and Molecular Function (MF) of up- and down-regulated genes in GO **(B)**. KEGG pathway classification of the top 20 differentially expressed genes **(C)**.

RNA-seq was conducted to examine the impact of HMB treatment on gene expressions in *F. graminearum*. The results shown in Figure 4A indicated that 1989 genes displayed differential expression, with 1,290 genes up-regulated and 699 genes down-regulated, while 11,327 genes showed no significant changes in expression. The GO enrichment analysis (Figure 4B) results categorized the differentially expressed genes (DEGs) into three groups: biological processes (BP), cellular components (CC), and molecular functions (MF). A total of 40 unique Gene Ontology (GO) categories (*p* < 0.05) were detected among the DEGs. In the context of Biological Processes (BP), the top three enriched categories were carbohydrate metabolic process, DNA-templated transcription, and rRNA processing. For Cellular Components (CC), the three most significantly represented categories were the integral component of the membrane, the cytoplasm, and the nucleus. For Molecular Functions (MF), the primary three categories were ATP binding, oxidoreductase activity, and transmembrane transporter activity. Among the top 40 annotated (KEGG) enrichment pathways, the first three were carbon metabolism, biosynthesis of amino acids and biosynthesis of cofactors (Figure 4C). As the content of ergosterol was reduced by HMB treatment, it is assumed that HMB treatment affects the expression of transcriptomic levels. Table 1 shows many genes participating in ergosterol biosynthesis, oxidative stress response, and DON biosynthesis were regulated. In detail, *Erg2*, *Erg5*, *Erg6*, *Erg7*, *Erg9*, *Erg24*, and *Erg27* in ergosterol biosynthesis were down-regulated. Among them, two genes with an identity of approximately 74%, located on different chromosomes, both encode sterol 24-C-methyltransferase (Erg6). Meanwhile, the expressions of genes encoding CAT, MnSOD, and Cu/ZnSOD enzymes in the redox system were down-regulated, whereas the expressions of GSS and HYR1 encoding enzymes were up-regulated. In addition, 9 genes were downregulated and 11 genes were upregulated in the DON biosynthesis pathway.

**Table 1:** Expression of genes participating in ergosterol biosynthesis, oxidative stress response, and DON biosynthesis of *F. graminearum* treated with HMB at 0 and 1/2 MIC.

	Gene name	Query ID	Log_2_	Style	*P* value	
Ergosterol biosynthesis	Farnesyl-diphosphate farnesyltransferas*e (Erg9)*	FGSG_09381	−0.83	down	2.37E-05	***
	Lanosterol s*ynthase (Erg7)*	FGSG_05950	−0.05	down	0.78	/
	Delta14-sterol reductase *(Erg24)*	FGSG_02346	−0.21	down	0.46	/
	3-keto steroid reductase *(Erg27)*	FGSG_13956	−0.15	down	0.66	/
	Sterol 24-C-methyltransferase *(Erg6)*	FGSG_02783	−0.02	down	0.93	/
	Sterol 24-C-methyltransferase *(Erg6)*	FGSG_05740	−0.1	down	0.84	/
	C-8 sterol isomerase *(Erg2)*	FGSG_07315	−0.28	down	0.31	/
	Sterol 22-desaturase *(Erg5)*	FGSG_03686	−0.05	down	0.91	/
Oxidative stress response	Mycelial catalase *(CAT)*	FGSG_02217	−1.66	down	2.30E-10	***
	Mn superoxide dismutase *(MnSOD)*	FGSG_04454	0.43	up	0.01	*
	Cu/Zn superoxide dismutase *(Cu/ZnSOD)*	FGSG_08721	0.87	up	1.00E-07	***
	*GSS*	FGSG_07268	−0.08	down	0.71	/
	*HYR1*	FGSG_06150	0.62	up	2.76E-03	**
	*LaeA*	FGSG_07660	0.91	up	3.20E-06	***
	*VelB*	FGSG_01362	0.58	up	1.43E-03	**
	*VeA*	FGSG_11955	−0.44	down	5.17E-03	**
	*FgSsk1*	FGSG_08948	0.31	up	0.07	/
	*FgSsk2*	FGSG_00408	0.03	up	0.84	/
	*FgPbs2*	FGSG_08691	0.47	up	0.06	/
	*FgHog1*	FGSG_09612	0.88	up	6.00E-07	***
Isoprenoid pathway	Mevalonate kinase	FGSG_05912	0.13	up	0.42	/
	Farnesyl diphosphate synthase	FGSG_06784	0.18	up	0.4	/
	Hydroxymethylglutaryl-CoA reductase *(NADPH)*	FGSG_09197	0.2	up	0.23	/
	Hydroxymethylglutaryl-CoA synthase	FGSG_09266	−0.06	down	0.74	/
	Acetyl-CoA C-acetyltransferase	FGSG_09321	0.47	up	2.37E-03	***
	Isopentenyl-diphosphate Delta-isomerase	FGSG_09722	−0.11	down	0.57	/
	Phosphomevalonate kinase	FGSG_09764	−0.14	down	0.45	/
	Diphosphomevalonate decarboxylase	FGSG_10424	0.19	up	0.3	/
Trichothecene pathway	Cytochrome P450 monooxygenase *(Tri1)*	FGSG_00071	0.24	up	0.46	/
	Trichothecene 15-O-acetyltransferase *(Tri3)*	FGSG_03534	2.13	up	0.67	/
	Cytochrome P450 monooxygenase *(Tri4)*	FGSG_03535	−0.08	down	0.96	/
	Trichodiene synthase *(Tri5)*	FGSG_03537	−1.84	down	0.71	/
	Trichothecene biosynthesis transcription regulator 6 *(Tri6)*	FGSG_03536	0.65	up	0.86	/
	Trichothecene C-3 esterase *(Tri8)*	FGSG_03532	−0.04	down	0.93	/
	*Tri9*	FGSG_03539	−0.6	down	0.57	/
	Trichothecene biosynthesis transcription regulator 10 *(Tri10)*	FGSG_03538	−0.19	down	0.89	/
	Trichothecene C-15 hydroxylase *(Tri11)*	FGSG_03540	/	/	/	/
	Trichothecene efflux pump *(Tri12)*	FGSG_02343	−0.15	down	0.69	/
	Core trichothecene cluster *(Tri14)*	FGSG_03543	0.35	up	0.77	/
	Trichothecene 3-O-acetyltransferase *(Tri101)*	FGSG_07896	0.42	up	0.05	/

Student’s t-test, *p < 0.05, **p < 0.01, ***p < 0.001, ****p < 0.0001.

### 3.4 HMB reduced the expression levels of genes in ergosterol biosynthesis

**Figure 5:**
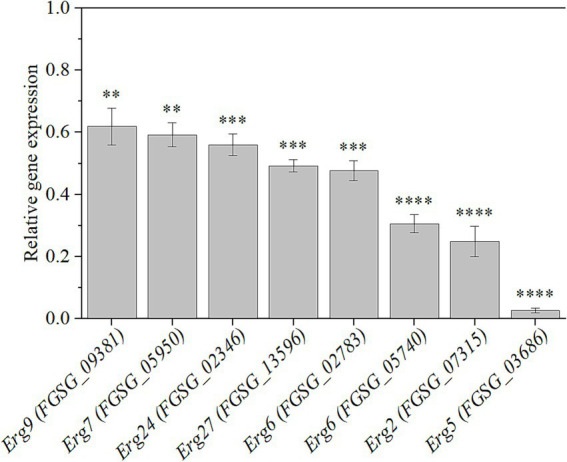
Relative expression levels of genes participating in ergosterol biosynthesis in response to HMB at 1/2 MIC. Black line represents normalized control expression at 1. Student’s *t*-test, **: *p* < 0.01, ***: *p* < 0.001, ****: *p* < 0.0001.

Ergosterol biosynthesis is an intricate process involving multiple enzymes (Supplementary Figure S1). The pathway begins with squalene synthase Erg9, which converts farnesyl pyrophosphate (FPP) into squalene, the key precursor for steroid synthesis. Squalene is then converted to lanosterol by squalene epoxidase Erg1 and lanosterol synthase Erg7 (Liu et al., 2019). Lanosterol undergoes a series of demethylation steps mediated by lanosterol C-14 demethylase Erg11 and the collaborative action of Erg24 to Erg27, producing zymosterol (Liu et al., 2011). Subsequently, zymosterol is converted into fecosterol through the catalyzation of C-24 methyltransferase Erg6. Fecosterol is then transformed into episterol by C-8 isomerase Erg2. In the final stages of the pathway, episterol is desaturated and reduced to ergosterol by Erg3, Erg5, and Erg4 (Han et al., 2023; Venegas et al., 2020). To confirm the expression changes of genes participating in ergosterol biosynthesis, qRT-PCR was conducted. As depicted in Figure 5, the expressions of *Erg9* (FGSG_09381), *Erg7* (FGSG_05950), *Erg24* (FGSG_02346), *Erg27* (FGSG_13956), *Erg6* (FGSG_02783, FGSG_05740), *Erg2* (FGSG_07315), and *Erg5* (FGSG_03686) genes were downregulated significantly (*p* < 0.01), confirming that HMB treatment inhibited ergosterol biosynthesis.

### 3.5 HMB downregulated expressions of genes participating in DON biosynthesis and genes encoding redox enzymes and regulatory factors

**Figure 6:**
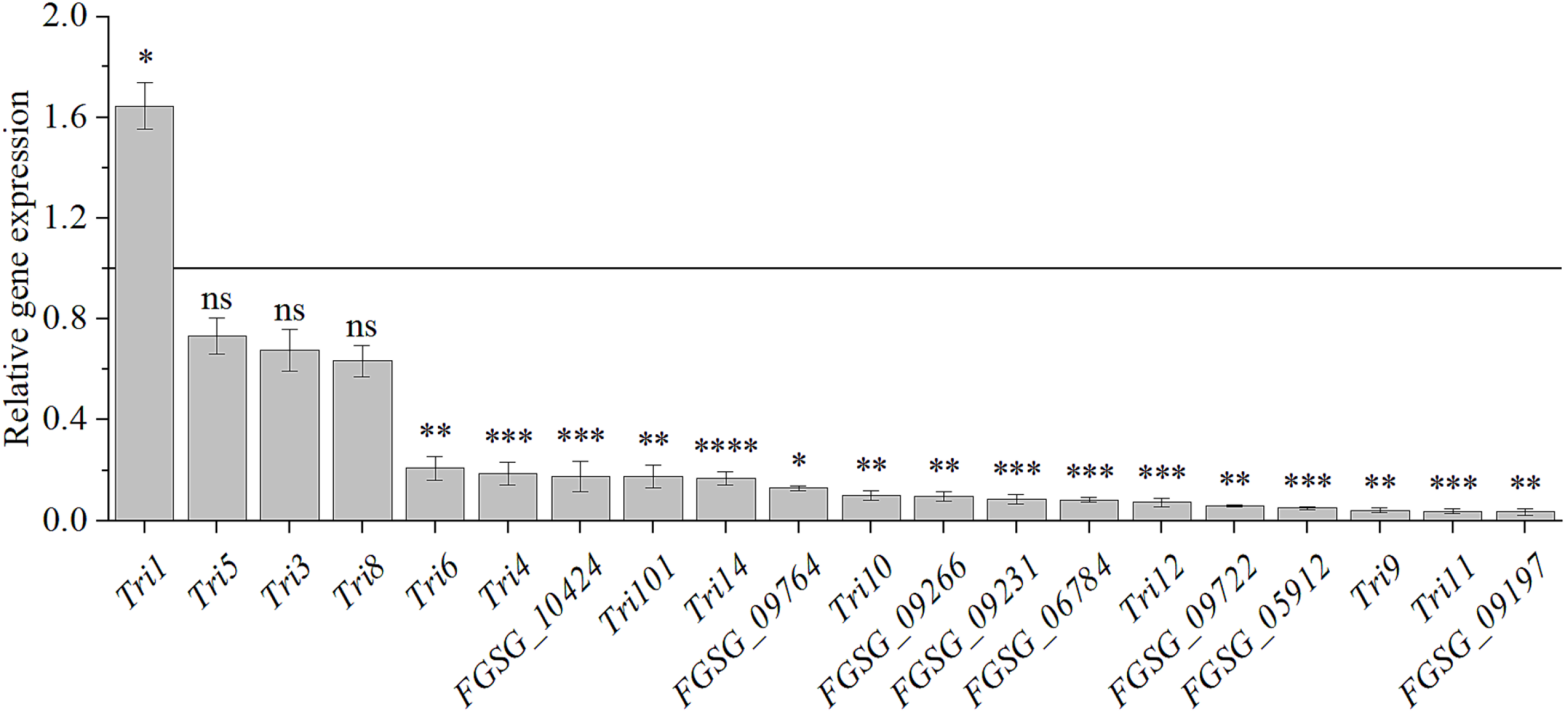
Relative expression levels of genes participating in DON biosynthesis in response to HMB at 1/2 MIC. Black line represents normalized control expression at 1. Student’s *t*-test, ns: not significant, *: *p* < 0.05, **: *p* < 0.01, ***: *p* < 0.001, ****: *p* < 0.0001.

As the biosynthesis of secondary metabolism was enriched in the RNA-seq analysis, we quantified the 20 DON biosynthetic genes expression after HMB treatment at 1/2 MIC by qRT-PCR. As illustrated in Figure 6, compared to the control group, *Tri1* gene was upregulated about 2 folds (*p* < 0.05). However, 20 genes were down-regulated, and among which, up to 16 genes were significantly down-regulated (*p* < 0.05); for the remaining three genes (*Tri5*, *Tri3*, and *Tri8*), they were indistinctively down-regulated.

**Figure 7:**
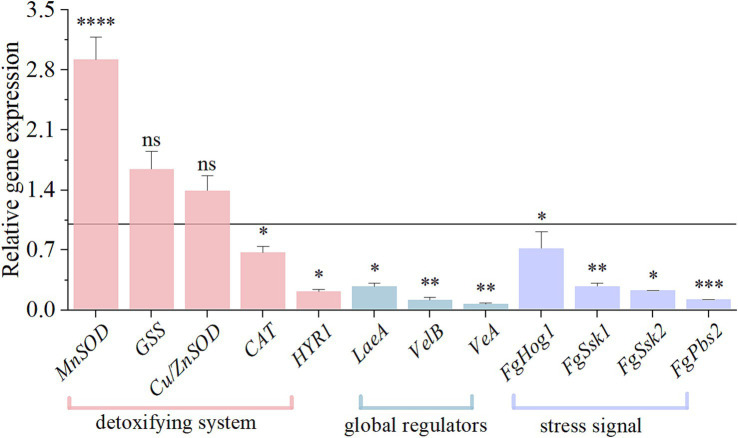
Effect of HMB on the relative expressions of genes involved in oxidative stress response in *F. graminearum*. Black line represents control expression level at 1. Student’s *t*-test, *: *p* < 0.05, **: *p* < 0.01, ***: *p* < 0.001, ****: *p* < 0.0001.

Previous studies have found that HMB treatment significantly elevated MDA and glycerol levels in *F. graminearum* (Li et al., 2024), leading to lipid oxidation and osmotic stress responses that may interfere with redox homeostasis and the HOG-MAPK pathway, in order to test this conjecture the expression of genes related to oxidative, HOG-MAPK, and velvet complexes (*LaeA*, *VelB*, and *VeA*) was analyzed. As depicted in Figure 7, after HMB treatment, *MnSOD* showed a notable 2.92-fold upregulation (*p* < 0.001) and *GSS* and *Cu/ZnSOD* were indistinctively up-regulated. The expressions of *CAT* and *HYR1* genes were decreased by 0.33-fold and 0.78-fold, respectively (*p* < 0.05). In addition, the expressions of global regulators (*LaeA*, *VelB*, and *VeA*) and 4 genes of the high osmolarity glycerol mitogen-activated protein kinase (HOG-MAPK) pathway (*FgSsk1*, *FgSsk2*, *FgPbs2*, and *FgHog1*) were markedly decreased (*p* < 0.05).

## 4 Discussion

Many plant-derived natural compounds are hydrophobic and prone to binding and disrupting cell membranes (Burt, 2004). The similar mode of action was also found in our publications (Li et al., 2021a; Li et al., 2021b; Li et al., 2021c; Li et al., 2020). Still, for HMB, the antifungal mechanism is still far from illustrated. Several commercial fungicides were developed as they could target ergosterol biosynthesis that is unique in fungi. For instance, azole fungicides (Ketoconazole, propiconazole, and tebuconazole) typically inhibits Erg11 activity (Qian et al., 2017). Allylamines and thiocarbamates target squalene epoxidase encoded by the *Erg1* gene, while morpholines (fenpropimorph and amorolfine) downregulated the expression of *Erg2* and *Erg24* genes (Prasad et al., 2016). In natural product fungistatic assays, substances that disrupt ergosterol biosynthesis often act on multiple genes. For example, citral reduces the expression of the *Erg7*, *Erg11*, *Erg6*, *Erg3*, and *Erg5* genes in *Penicillium digitatum* (OuYang et al., 2016), while cinnamaldehyde downregulates the gene expression of *Erg11*, *Erg6*, and *Erg4* in *Fusarium sambucinum* (Wei et al., 2020). Thymol interferes with ergosterol biosynthesis in *F. graminearum* in a manner similar to azoles (Gao et al., 2016). In our present study, HMB treatment suppressed the expression of 8 genes in the ergosterol biosynthesis pathway, including *Erg9* (FGSG_09381), *Erg7* (FGSG_05950), *Erg24* (FGSG_02346), *Erg27* (FGSG_13956), *Erg6* (FGSG_02783 and FGSG_05740), *Erg2* (FGSG_07315), and *Erg5* (FGSG_03686). Among them, the most strongly downregulated genes were located downstream, including *Erg6* (FGSG_05740), *Erg2*, and *Erg5*. Hence, HMB inhibits the growth of *F. graminearum* by disrupting the cell membrane through downregulating the genes participating in ergosterol biosynthesis.

Under normal physiological conditions, ROS is generated through routine metabolic processes, with its production and scavenging maintained in a delicate balance (Dwyer et al., 2014). Excessive ROS accumulation could ultimately reduce cell viability and potentially cause cell death (Chen et al., 2023; Hwang et al., 2012). In this study, HMB treatment resulted in ROS accumulation, accompanied by increased SOD activity, decreased CAT activity, and unchanged GPx activity. This suggested that while the activity of SOD was boosted to counteract elevated ROS levels, the activities of other antioxidant enzymes (GPx and CAT) either remained unchanged or decreased, leading to sustained high intracellular ROS levels. In addition, as GSH levels remained relatively stable in response to HMB treatment, the non-enzymatic antioxidant is supposed to contribute little to ROS scavenging. Consistent with our hypotheses, H₂O₂ levels increased, likely caused by the differential regulations of these antioxidant enzymes, further contributing to ROS accumulation. Similar changes in enzyme activities and H_2_O_2_ levels were observed in *A. flavus* treated with paeonol (Li et al., 2022). However, other compounds exhibited varying effects on antioxidant enzymes besides causing ROS accumulation. For instance, methyl gallate reduced the activities of SOD, CAT, and POD (Liu et al., 2021), whereas *α*-thujone treatment increased CAT activity in *F. graminearum* (Teker et al., 2021). In addition, 4-propylphenol exposure significantly reduced GSH and GSSH levels in *F. graminearum* (Sun et al., 2024). In *Candida glabrata*, fluconazole treatment increased the GPx and SOD activity (Mahl et al., 2015). Therefore, redox systems in different fungi respond differently upon various compounds treatments.

DON biosynthesis occurs in two stages (Supplementary Figure S2): the first one is the isoprenoid pathway, which starts with acetyl-CoA and involves 7 reactions catalyzed by enzymes such as acetoacetyl-CoA synthase and HMG-CoA synthase. The final step in this pathway is the conversion of mevalonic acid to FPP. The second one is the trichothecene pathway, which involves 15 enzyme-mediated reactions converting FPP to DON. Initially, FPP is cyclized by Tri5 encoding trichodiene synthase to form trichodiene (TDN). After a series of catalyzation by the multifunctional cytochrome P450 monooxygenase (Tri4), isotrichotriol is formed (Oufensou et al., 2020). Then, Isotrichotriol undergoes non-enzymatic isomerization and cyclization to form isotrichodermol. Subsequently, Tri101, Tri11 and Tri3 then catalyze the formation of the first potentially toxic substance, calonectrin (CAL) (McCormick et al., 2011). In the last step, DON was produced by the catalyzation of Tri8 (McCormick et al., 2011). *Tri6* and *Tri10* are key regulatory genes in the production of DON (Seong et al., 2009). In detail, Tri6, a Cys2-His2-type transcription factor, regulates the *Tri* gene cluster consisting of *Tri5*, *Tri12*, and *Tri4*. As an upstream regulator, *Tri10* indirectly controls DON biosynthesis by modulating the expression of downstream genes, such as *Tri4* and *Tri5*, through the regulation of *Tri6* (Ponts et al., 2007). Among the 16 downregulated DON biosynthesis genes, all 8 genes of the isoprenoid pathway were significantly down-regulated. Six genes (*Tri4*, *Tri9*, *Tri11*, *Tri12*, *Tri14*, and *Tri101*) located in the middle part of the biosynthetic pathway were significantly down-regulated except for *Tri3*, which was not significantly changed. *Tri1* was significantly up-regulated, *Tri5* and *Tri8* genes, located at the beginning and end of the pathway, were not significantly regulated. For the two transcription factors, *Tri6* and *Tri10*, they were also significantly down-regulated. Similarly, other compound treatments downregulated genes in the *Tri* gene cluster. For example, ferulic acid treatment downregulated the expressions of *Tri4*, *Tri5*, *Tri6*, *Tri11*, *Tri12*, *Tri101*, and *Tri10* genes (Boutigny et al., 2009), while methyl gallate (MG) treatment downregulated the expressions of *Tri3*, *Tri5*, *Tri8*, *Tri10*, *Tri11*, and *Tri12* genes (Liu et al., 2021). The downregulation of *Tri5* gene was also found after myrcene treatment (Albayrak et al., 2023). However, the expression changes of other genes in the DON biosynthesis pathway were not mentioned in these papers.

Interestingly, our recent study showed that MDA content was significantly negatively correlated with DON content in *F. graminearum* after HMB treatment (Li et al., 2024). In addition, at the transcriptome level, mycotoxin production decreasing seems to be accompanied by the expression changes of redox enzyme genes. For instance, ethanol has been shown to inhibit the biosynthesis of aflatoxin B_1_ (AFB_1_), meanwhile, redox enzyme genes (*Cat*, *Cat1*, *Cat2*, *CatA*, and Cu, Zn superoxide dismutase gene *SOD1*) were upregulated and *MnSOD* gene was downregulated (Ren et al., 2020). Cinnamaldehyde treatment suppressed AFB_1_ production, and also upregulated the expressions of *Cat1*, *CatA*, *SOD1*, and *MnSOD* genes in *A. flavus* (Wang et al., 2019). Furthermore, other works have proved that several such genes are upstream regulators of DON biosynthesis. For example, knockout of the *Cu/ZnSOD* gene induced a significant decrease in DON production (Yao et al., 2016). Two mutants of *SOD2* and *SOD3* genes remarkably downregulated *Tri5* and *Tri6* genes expression and reduced 3-DON production (Furukawa et al., 2017). In the present work, HMB treatment up-regulated the expressions of *MnSOD*, *GSS*, and *Cu/ZnSOD* genes, while *CAT* and *HYR1* genes were down-regulated. The gene expression results were consistent with the above enzyme activity results. Still, whether other redox enzymes (e.g., CAT, GSS, and HYR1) regulate DON biosynthesis requires further knockout and/or overexpression works.

Apart from redox enzymes, global regulators are known to regulate the DON biosynthetic gene cluster (Chen et al., 2019). Light is known to positively regulate DON biosynthesis in *F. graminearum* via specific regulation of the velvet complex (VelB/VeA/LaeA) (Chen et al., 2019), similar as those in *A. flavus* (Amare and Keller, 2014). In detail, the individual deletion of each of the three genes significantly downregulated the expressions of *Tri5* and *Tri6* genes (Jiang et al., 2012; Merhej et al., 2011; Yu et al., 2013). What’s more, according to the significantly elevated glycerol content in *F. graminearum* on exposure of HMB treatment, we supposed that the HOG-MAPK pathway was regulated, as this pathway responds to osmotic stress (Ochiai et al., 2007). Previous researches showed that several genes participating in HOG-MAPK pathway could regulate DON biosynthesis. In detail, three knockout mutants of *FgSsk2*, *FgPbs2*, and *FgHog1 gene* downregulated the transcript levels of *Tri4* and *Tri6* genes (Ochiai et al., 2007; Zheng et al., 2012). In this work, HMB treatment significantly downregulated the velvet complex genes (*VelB*, *VeA*, and *LaeA*) and HOG-MAPK pathway-related genes (*FgSsk1*, *FgSsk2*, *FgPbs2*, and *FgHog1*), indicating a collective contribution on the inhibition of DON biosynthesis.

## 5 Conclusion

As chemical fungicides are potential threats to environment (e.g., soil residues, resistance), human and animal health, green fungicide development is driven. In this study, the antifungal mechanism of HMB against *F. graminearum* was further elucidated. HMB inhibits mycelial growth by targeting the ergosterol biosynthesis, the anti-mycotoxigenic mechanism is probably involved with the participation of redox enzymes, velvet complex, and key factors in HOG-MAPK pathway. However, *F. graminearum* has diverse strains with varying physiological and pathogenic traits. Their sensitivity to compounds differs, so the inhibitory effects of HMB on other strains, apart from PH-1, need further study. Also, new transcriptional regulators, the relationship between redox system and the DON biosynthesis pathway still requires to be explored and elucidated. Based on these efforts and results, HMB holds a great promise in developing as an efficient fungicide in the preservation of agricultural products and food.

## Data Availability

The raw data supporting the conclusions of this article will be made available by the authors, without undue reservation.

## Footnotes

^1^ https://biosys.bgi.com

## Supplementary material

SUPPLEMENTARY FIGURE S1 Ergosterol biosynthesis pathway.

SUPPLEMENTARY FIGURE S2 DON biosynthesis pathway.

SUPPLEMENTARY TABLE S1 Primer sequences used in qRT-PCR.
