# Supplementary figures and images for: 2-Hydroxy-4-methoxybenzaldehyde (HMB) disrupts ergosterol biosynthesis, redox metabolism, and DON biosynthesis of Fusarium graminearum revealed by transcriptome analysis
Source: Front Microbiol. 2025 Jun 2;16:1514170. doi: 10.3389/fmicb.2025.1514170 (PMC12171200; doi:10.3389/fmicb.2025.1514170)

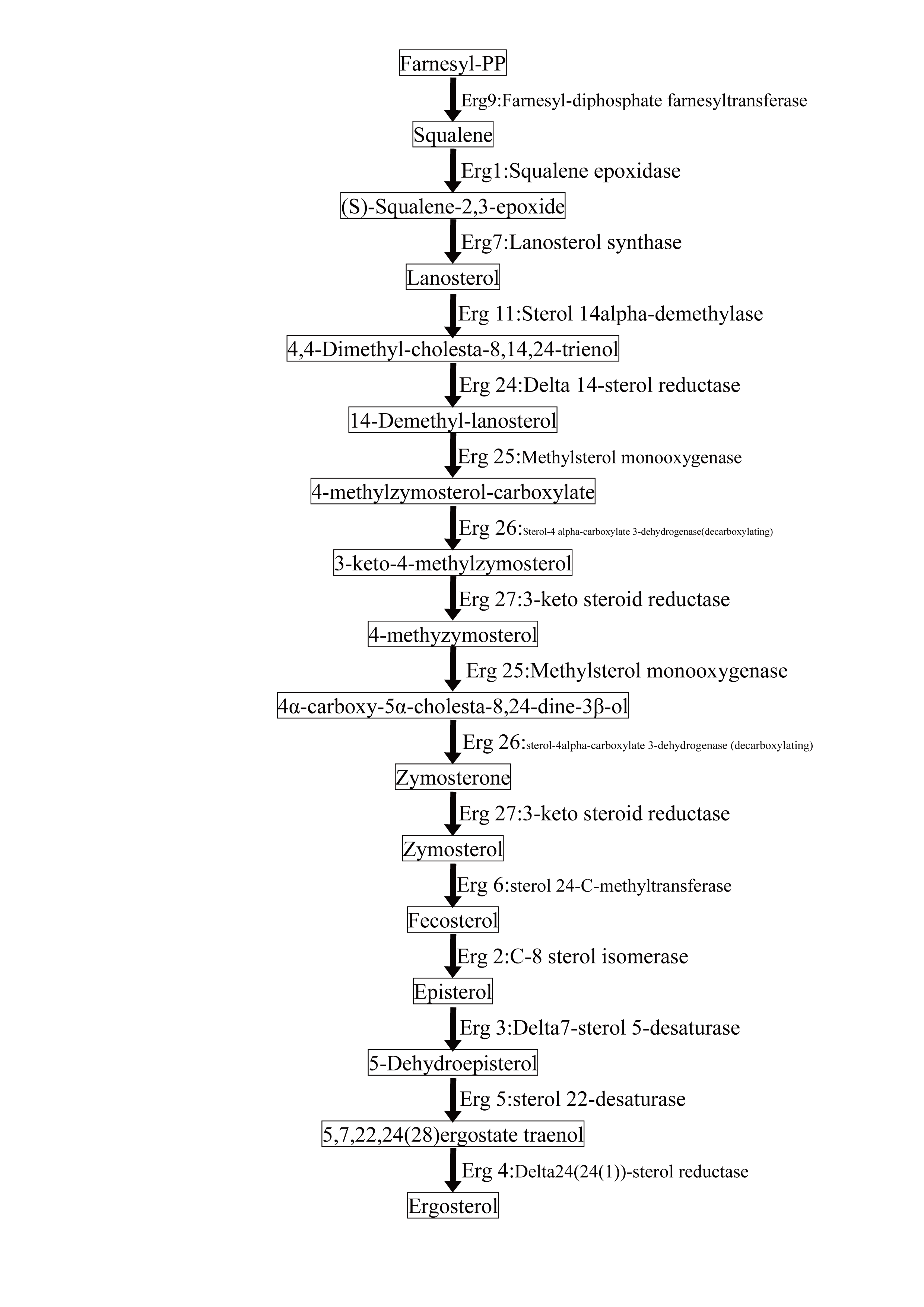

Supplement: SUPPLEMENTARY FIGURE S1 — Ergosterol biosynthesis pathway. [file Image_1.tif]

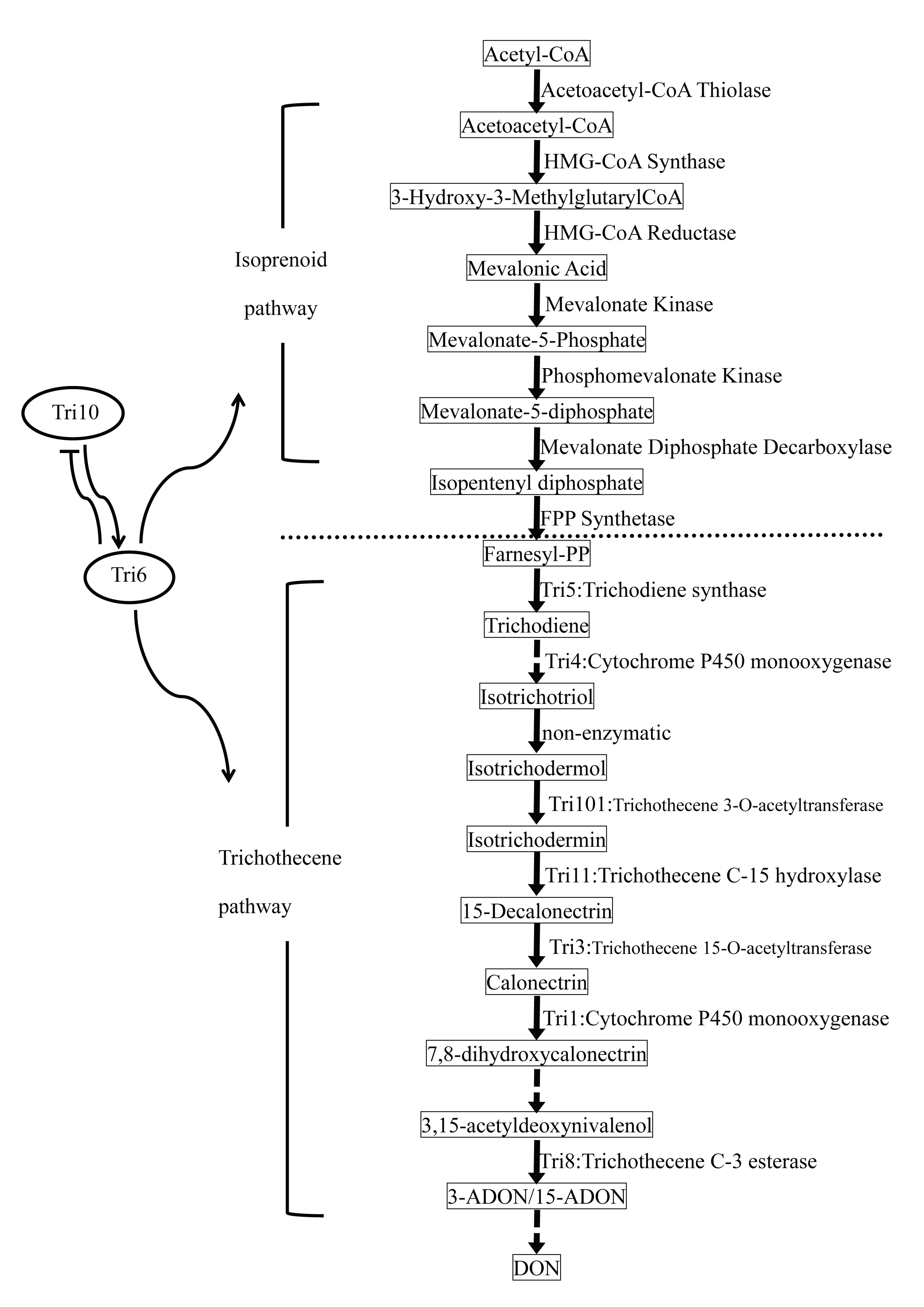

Supplement: SUPPLEMENTARY FIGURE S2 — DON biosynthesis pathway. [file Image_2.tif]
