# Supplementary material for: 2-Hydroxy-4-methoxybenzaldehyde (HMB) disrupts ergosterol biosynthesis, redox metabolism, and DON biosynthesis of Fusarium graminearum revealed by transcriptome analysis
Source: Front Microbiol. 2025 Jun 2;16:1514170. doi: 10.3389/fmicb.2025.1514170 (PMC12171200; doi:10.3389/fmicb.2025.1514170)
Supplement: Supplementary file 3 [file Data_Sheet_1.docx]

Table S1 Primer sequences used in qRT-PCR

| Gene Name | Primer sequence (5’-3’forward/reverse) |
| --- | --- |
| FGSG_09381 (*Erg9*) | CTACGCCGAGAACGAGGAGATG TTCACCGACGAGACCAGCAAC |
| FGSG_05950 (*Erg7*) | AAGCAGCCTCGCATCGGTTC CAGCATCGTCGTCCTCAAGGTAG |
| FGSG_02346 (*Erg24*) | GGTCTCTACATCTTCCGCTCTTCC CCACCAGCCGCCTGTCAAG |
| FGSG_13956 (*Erg27*) | GGCACAAACATCAGGCTCCATAC  ACGAGGAACGAAGTCAGGTGTC |
| FGSG_02783 (*Erg6*) | AGTCCTTCCACTTCTGCCGATTC CGCAACCGACATCAAGAACCTTC |
| FGSG_05740 (*Erg6*) | CGTTGGATAGTGACATGCGATATGC CAGCCGTCTTTCTTGTTCCTTTCG |
| FGSG_07315 (*Erg2*) | AAAGTCGCAGGGTGGCATCAG GATCGTCGAGGTGCTTGAGGTC |
| FGSG_03686 (*Erg5*) | TTCCAATGGCAGAGACTCTCCTTC GTCCTCGGCGGTATTCGGTATG |
| FGSG_02217 (*CAT*) | CAGCCTTTGCGTCCTTCCTTG ATATGTGATGGTGCCGTAGTTGTG |
| FGSG_04454 (*MnSOD*) | ACAACGCTACCGACGCCATC CCGTGGAAGTTGAGGAGAGGAG |
| FGSG_08721 (*Cu/ZnSOD*) | GCATAGCGAACCGTTGTCATCC ACCAGCGTTACCAGTCTTGAGAG |
| FGSG_07268 (*GSS*) | AGGAAAGGTTCATCACAACGAAGAG CCGAAGCCAGCAGCAACAC |
| FGSG_06150 (*HYR1*) | CCGACGATGACATTCAGGAGTTC CTTGAGCCAGACCCAGAGAGG |
| FGSG_07660 (*LaeA*) | CTGGCTGAGGAGACTGACTGAC CTCGGCATAGGTCCATTCATCATC |
| FGSG_01362 (*VelB*) | GCGAAGCGAAGTAGCCATCAG ACGAAGCGAGGAGGAGAAGAAG |
| FGSG_11955 (*VeA*) | CAACGCCCACCACCAACAAC TTCGGATGGTCGGTCAAGATAGG |
| FGSG_08948 (*FgSsk1*) | AACACTTCTCAAGGCTACCACATTC CGGCGTCGGCAGAGGAG |
| FGSG_00408 (*FgSsk2*) | GGTTGCTGGATGTATGTCTCTTCTC TGCCTCAATCCGCTCCTTCTC |
| FGSG_08691 (*FgPbs2*) | TGAATCCGAACTCTACTCCGATAGG TCCACCGAGGCACTACTGTTTC |
| FGSG_09612 (*FgHog1*) | TCTATCCCACCACACTCACACTATC TCGTCTTCCTTAGTCTTCTCGTCTC |
| FGSG_00071 (*Tri1*) | GAAGATTCCTGAAGGTCCCG TGTACCAATTCCAATCGCAGAC |
| FGSG_02343 (*Tri12*) | ATCCCCGCAGTCATTCAGA CGCAATCATAGCCACTACACC |
| FGSG_03532 (*Tri8*) | ATTTACCAAGCTATCCACGACG CACTGAAAAGCCGCCTCAT |
| FGSG_03534 (*Tri3*) | ACGGTGTCAACGAACGCTT TCCAGTCGGATTGCCAGATA |
| FGSG_03535 (*Tri4*) | ATGGCCTTTGCTGAGATGTACC CCAACAATACGGGCGTGAGT |
| FGSG_03536 (*Tri6*) | ACCGTGCTCGGCATGAGT TCCACCCTGCTAAAGACCCT |
| FGSG_03537 (*Tri5*) | AGCTCACCCAGGAAACCCT CACTCAATCGTGTCCATCACC |
| FGSG_03538 (*Tri10*) | TGGGCTCGACAAGACATTT CCATCCCTCAACCAAGACA |
| FGSG_03539 (*Tri9*) | TTGGCTGCGACCCATATG GCCGCTAAATTGATCGACTC |
| FGSG_03540 (*Tri11*) | TTCACCCGACCAAACGACT TCTGCGTAGGCAAGGTTCAT |
| FGSG_03543 (*Tri14)* | TCTCCAGAACGCCTTGACG CGAACCTGCTGCTCTTACCG |
| FGSG_07896 (*Tri101*) | CGCCAGCGAACAAGAGGT AAAGTCGTAATCCCAGAGTCCC |
| FGSG_05912 | GGCGGCTGCTCCATTACC CACCTCCAAGTGTCGTCTCAAA |
| FGSG_06784 | AAGTGCTCTTGGCTCGTGAA CCAGCTCCTTGGCATCGT |
| FGSG_09197 | TCGGATACATGCCCCTCC CAAGGACCACGAGTCATACCAT |
| FGSG_09266 | CCACCAACTGCGGTAACATG GATGCGGAAAGACAGGAAACT |
| FGSG_09321 | CTGGCTATTCCCAAGGCTATC GCAACGGAACCACCGTAGA |
| FGSG_09722 | TCTCACCCTCTTCACATCCCT CTCACCCCACATTCCGTCA |
| FGSG_09764 | TCCCTGTGACCCTGAGCAA AAGGAGCGAGGCGGTTAGT |
| FGSG_10424 | GACACCTACCCTCCCATCTTCT ATCAGCGTCCTTCTCCTCGT |
| β-tubulin | CGTCCAGAGCAAGAACTCATCA TGCGTCGGAACATAGCAGTAA |
